# Supplementary material for: Structure of the Newcastle Disease Virus L protein in complex with tetrameric phosphoprotein
Source: Nat Commun. 2023 Mar 10;14:1324. doi: 10.1038/s41467-023-37012-y (PMC10006412; doi:10.1038/s41467-023-37012-y)
Supplement: Supplementary file 1 — Supplementary information [file 41467_2023_37012_MOESM1_ESM.pdf]

Supplementary information

Structure of the Newcastle Disease Virus L protein in complex with tetrameric phosphoprotein

J. Cong *et al.*

a

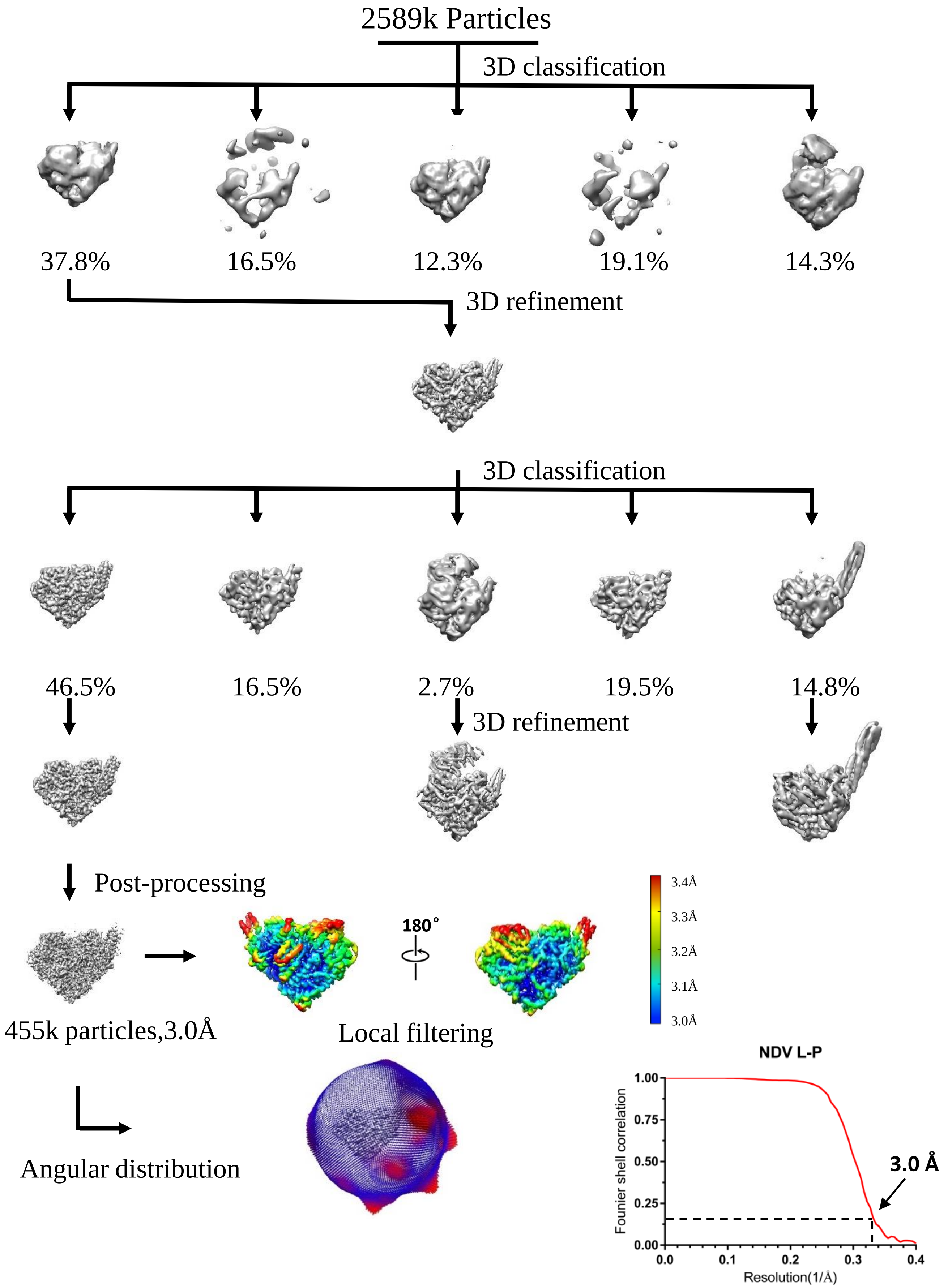
2589k Particles
3D classification
37.8%
16.5%
12.3%
19.1%
14.3%
3D refinement
3D classification
46.5%
16.5%
2.7%
19.5%
14.8%
3D refinement
Post-processing
180°
3.4Å
3.3Å
3.2Å
3.1Å
3.0Å
455k particles,3.0Å
Local filtering
Angular distribution
NDV L-P
Fourier shell correlation
Resolution(1/Å)
3.0 Å

b

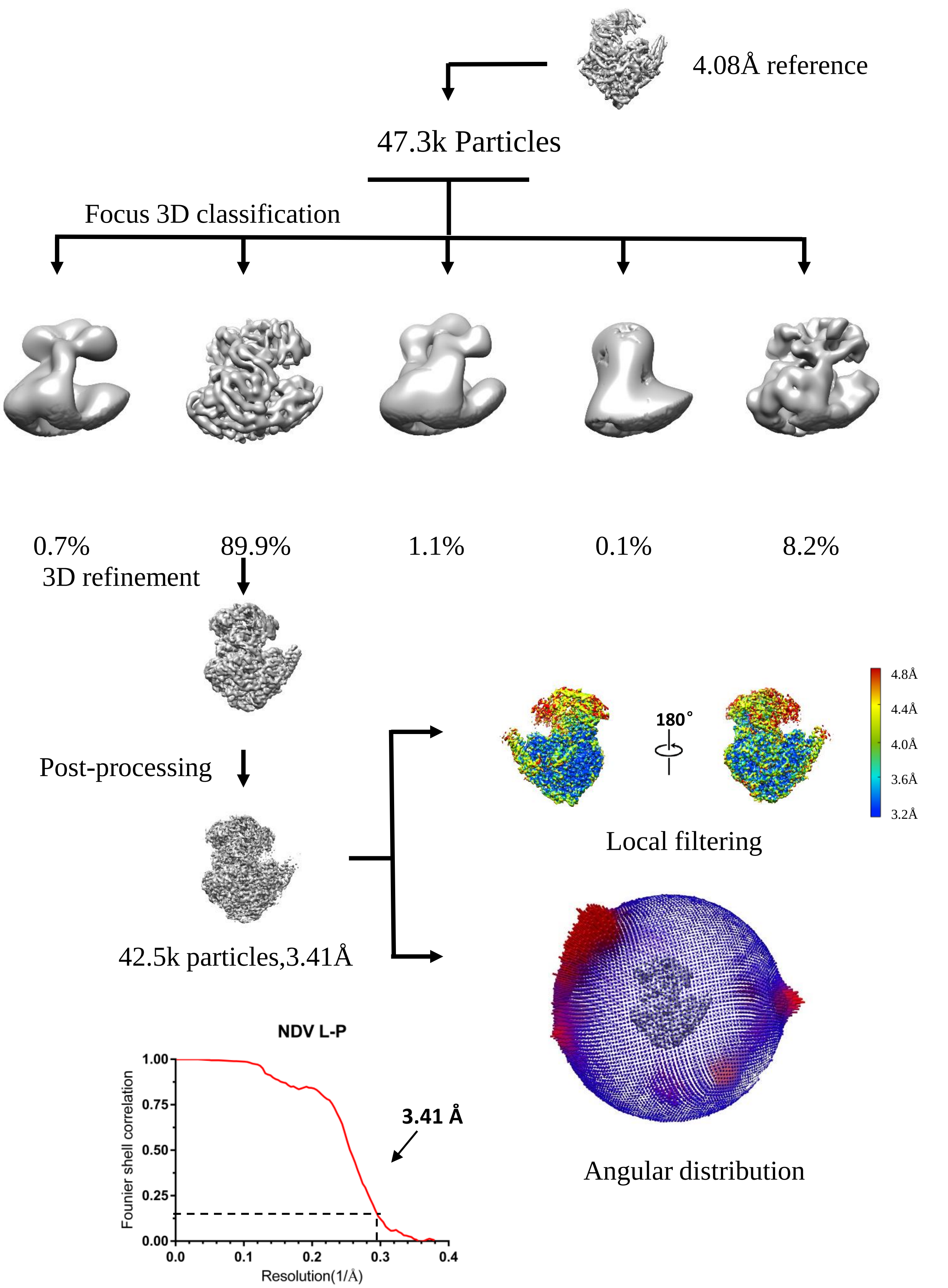
4.08Å reference
47.3k Particles
Focus 3D classification
0.7%
89.9%
1.1%
0.1%
8.2%
3D refinement
Post-processing
42.5k particles,3.41Å
180°
4.8Å
4.4Å
4.0Å
3.6Å
3.2Å
Local filtering
Angular distribution
NDV L-P
Founier shell correlation
Resolution(1/Å)
3.41 Å
1.00
0.75
0.50
0.25
0.00
0.0
0.1
0.2
0.3
0.4

c

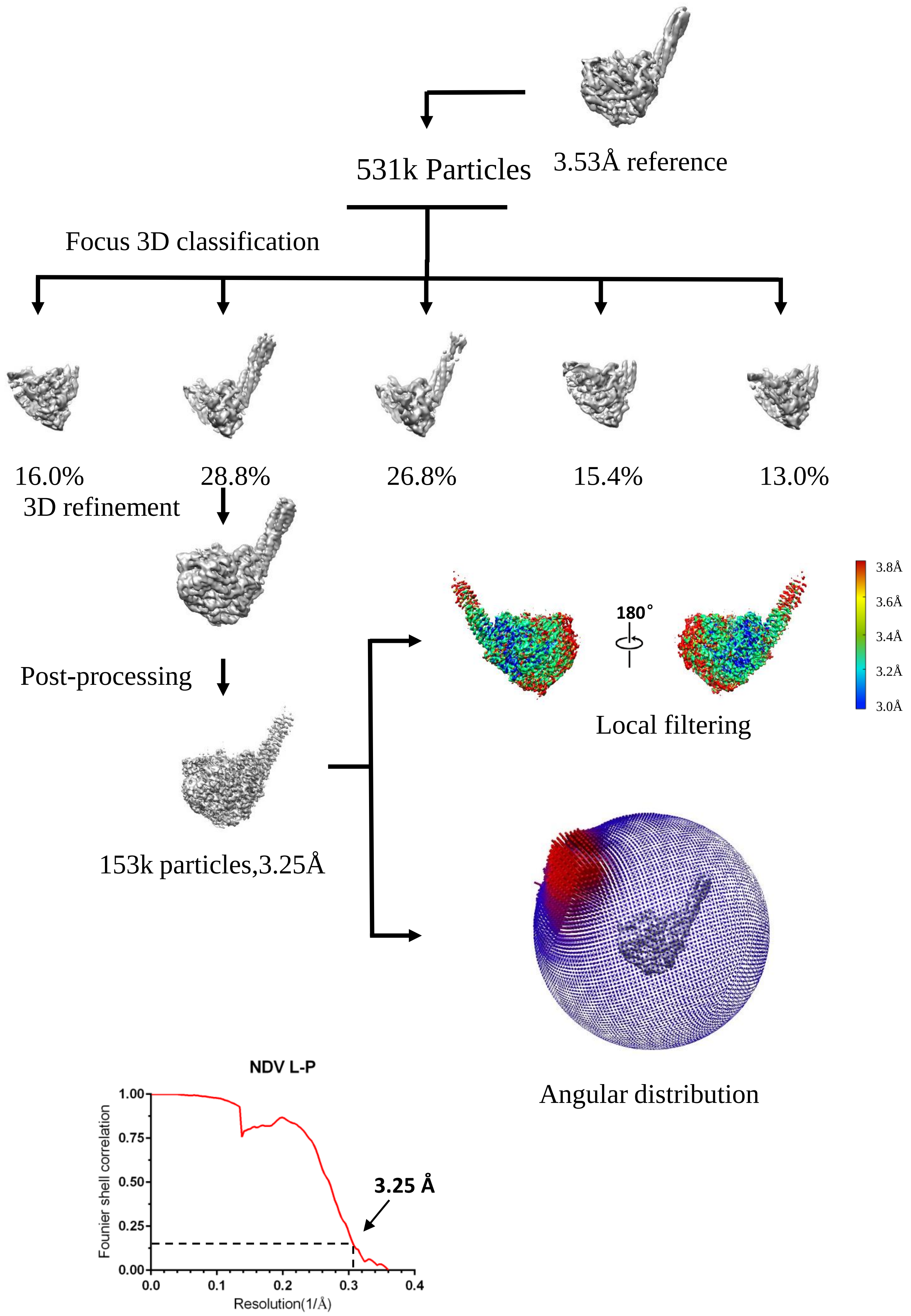

3.53Å reference
531k Particles
Focus 3D classification
16.0%
28.8%
26.8%
15.4%
13.0%
3D refinement
Post-processing
153k particles,3.25Å
180˚
Local filtering
3.8Å
3.6Å
3.4Å
3.2Å
3.0Å
Angular distribution
NDV L-P
Founier shell correlation
1.00
0.75
0.50
0.25
0.00
0.0
0.1
0.2
0.3
0.4
Resolution(1/Å)
3.25 Å

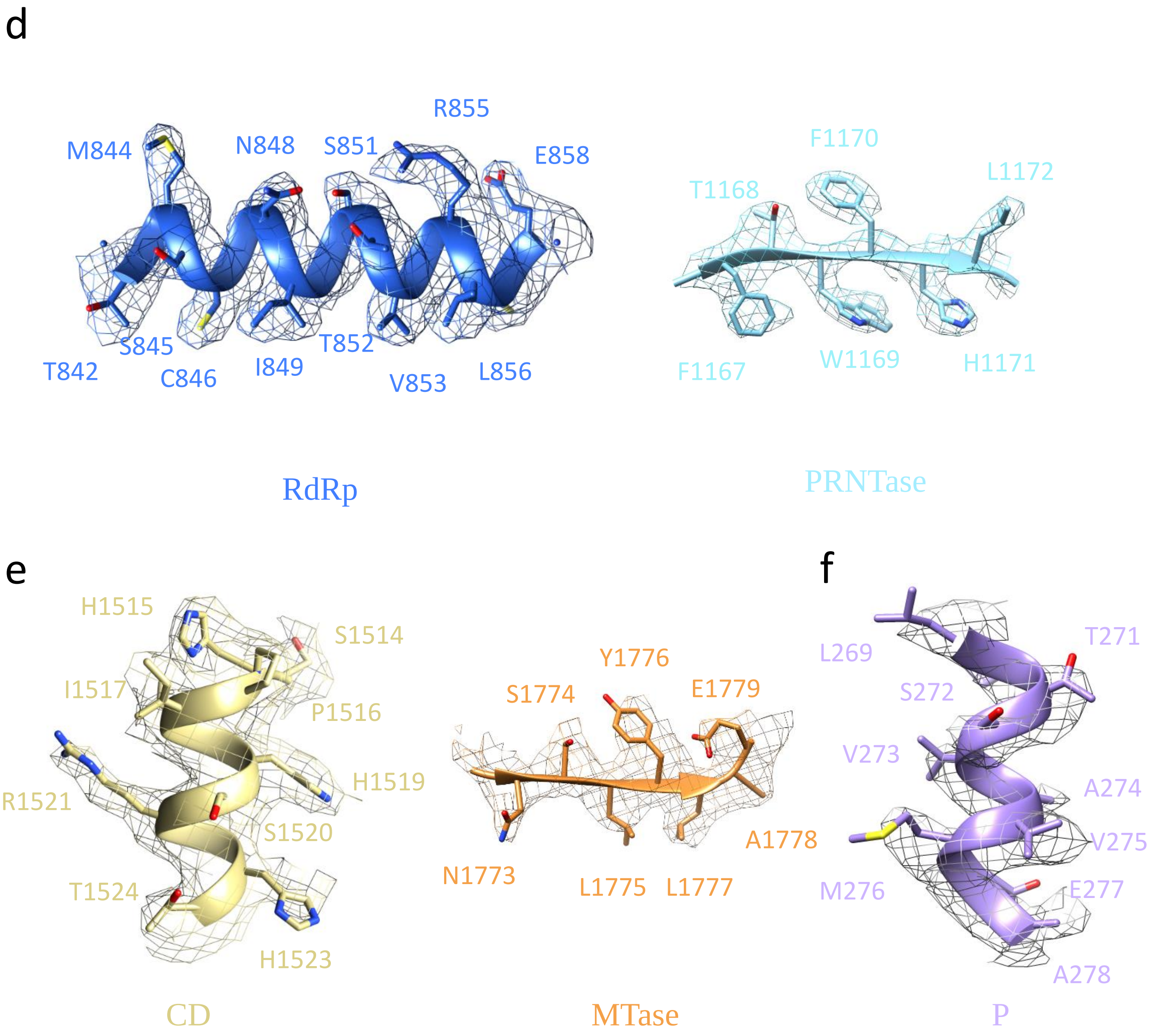

Supplementary Figure 1: Cryo-EM analysis of the NDV L-P complex. a, Brief 3D classification of NDV L_c-P complex particles. We extracted approximate 2,589k particles, and after two rounds of 3D classification, obtained approximate 455k particles for further 3D reconstruction, with the final map reaching a resolution of 3.0Å. The initial map of NDV L_f-P and L_c-P_e were 4.08 Å and 3.53 Å respectively. Local resolution and euler angle distribution of NDV L_c-P reconstruction were demonstrated at the bottom. **b,** Brief 3D classification of NDV L_f-P complex particles. We extracted approximate 47.3k particles, and after 3D classification, obtained approximate 42.5k particles for further 3D reconstruction, with the final map reaching a resolution of 3.41Å. Local resolution and euler angle distribution of NDV L_f-P reconstruction were demonstrated at the bottom. **c,** Brief 3D classification of NDV L_c-P_e complex particles. We extracted approximate 531k particles, and after 3D classification, obtained approximate 153k particles for further 3D reconstruction, with the final map reaching a resolution of 3.25Å. Local resolution and euler angle distribution of NDV L_c-P_e reconstruction were demonstrated at the bottom. **a,b,c,** The FSC curves for each reconstruction. The FSC 0.143 cut-off values are indicated by black dashed lines. **d,e,f,** Representative density of three NDV L-P complexes reconstructions.

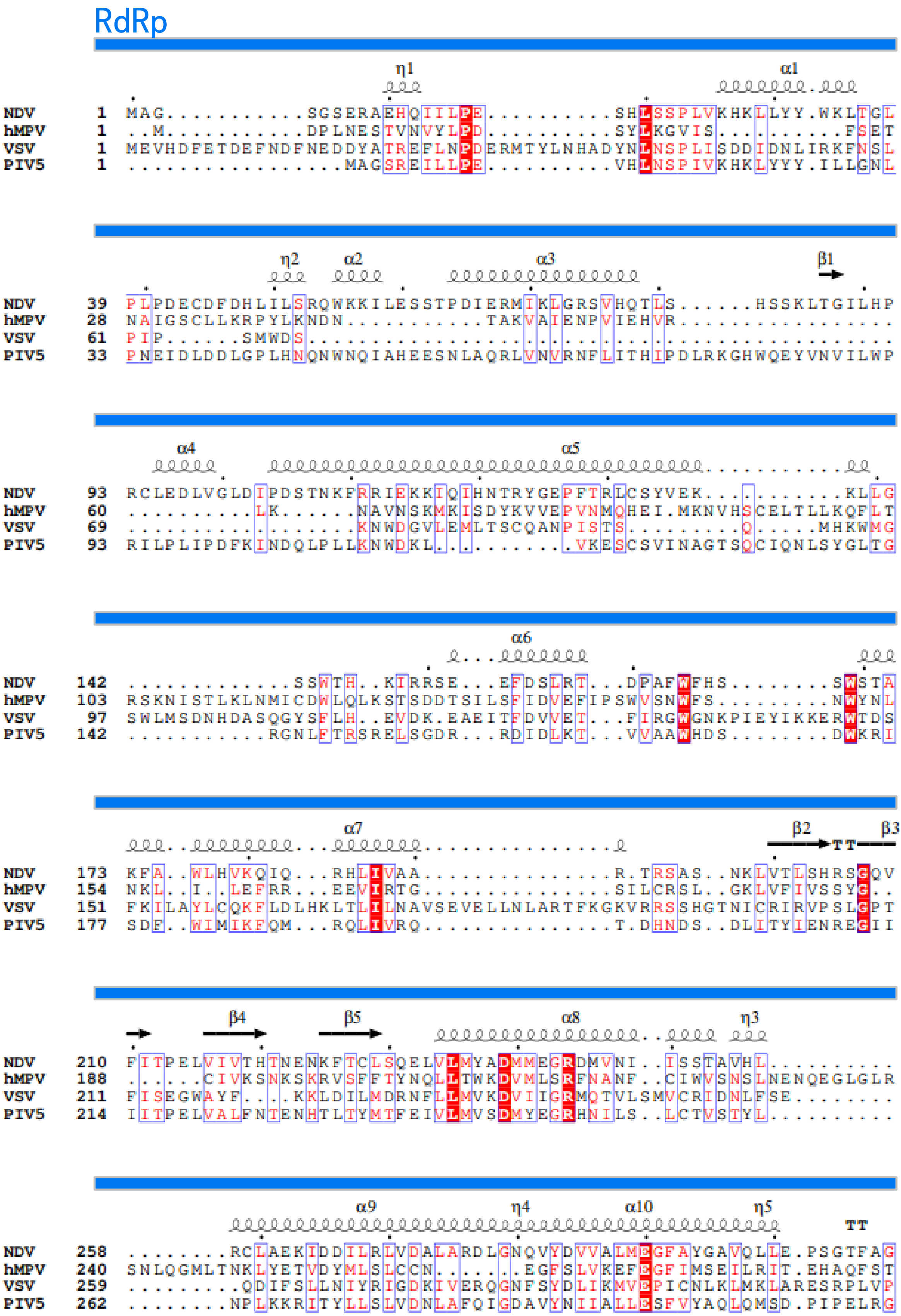
RdRp
η1 α1
NDV 1 MAG..........SGSERAEHQIILPE..........SHLSSPLVKHKLLYY.WKLTGL
hMPV 1 ..M..........DPLNESTVNVYLPD..........SYLKGVIS...........FSET
VSV 1 MEVHDFETDEFNDFNEDDYATREFLNPDERMTYLNHADYNLNSPLISDDIDNLIRKFNSL
PIV5 1 .................MAGSREILLPE..........VHLNSPIVKHKLYYY.ILLGNL
η2 α2 α3 β1
NDV 39 PLPDECDFDHLILSRQWKKILESSTPDIERMIKLGRSVHQTLS......HSSKLTGILHP
hMPV 28 NAIGSCLLKRPYLKNDN...........TAKVAIENPVIEHVR.................
VSV 61 PIP.....SMWDS...
PIV5 33 PNEIDLDDLGPLHNQNWNQIAHEESNLAQRLVNVRNFLITHIPDLRKGHWQEYVNVILWP
α4 α5
NDV 93 RCLEDLVGLDIPDSTNKFRRIEKKIQIHNTRYGEPFTRLCSYVEK...........KLLG
hMPV 60 ..........LK......NAVNSKMKISDYKVVEPVNMQHEI.MKNVHSCELTLLKQFLT
VSV 69 ..................KNWDGVLEMLTSCQANPISTS.........Q.....MHKWMG
PIV5 93 RILPLIPDFKINDQLPLLKNWDKL.........VKESCSVINAGTSQCIQNLSYGLTG
α6
NDV 142 ..............SSWTH..KIRRSE...EFDSLRT...DPAFWFHS........SWSTA
hMPV 103 RSKNISTLKLNMICDWLQLKSTSDDTSILSFIDVEFIPSWVSNWFS.........NWYNL
VSV 97 SWLMSDNHDASQGYSFLH..EVDK.EAEITFDVVET...FIRGWGNKPIEYIKKERWTDS
PIV5 142 ...........RGNLFTRSRELSGDR...RDIDLKT...VVAAWHDS........DWKRI
α7 β2 TT β3
NDV 173 KFA..WLHVKQIQ...RHLIVAA...............R.TRSAS..NKLVTLSHRSGQV
hMPV 154 NKL..I..LEFRR...EEVIRTG...............SILCRSL..GKLVFIVSSYG..
VSV 151 FKILAYLCQKFLDLHKLTLILNAVSEVELLNLARTFKGKVRRSSHGTNICRIRVPSLGPT
PIV5 177 SDF..WIMIKFQM...RQLIVRQ...............T.DHNDS..DLITYIENREGII
β4 β5 α8 η3
NDV 210 FITPELVIVTHTNENKFTCLSQELVLMYADMMEGRDMVNI..ISSTAVHL..........
hMPV 188 ......CIVKSNKSKRVSFFTYNQLLTWKDVMLSRFNANF..CIWVSNSLNENQEGLGLR
VSV 211 FISEGWAYF....KKLDILMDRNFLLMVKDVIIGRMQTVLSMVCRIDNLFSE........
PIV5 214 IITPELVALFNTENHTLTYMTFEIVLMVSDMYEGRHNILS..LCTVSTYL..........
α9 η4 α10 η5 TT
NDV 258 ........RCLAEKIDDILRLVDALARDLGNQVYDVVALMEGFAYGAVQLLE.PSGTFAG
hMPV 240 SNLQGMLTNKLYETVDYMLSLCCN.......EGFSLVKEFEGFIMSEILRIT.EHAQFST
VSV 259 .........QDIFSLLNIYRIGDKIVERQGNFSYDLIKMVEPICNLKLMKLARESRPLVP
PIV5 262 ........NPLKKRITYLLSLVDNLAFQIGDAVYNIIALLESFVYAQLQMSD.PIPELRG

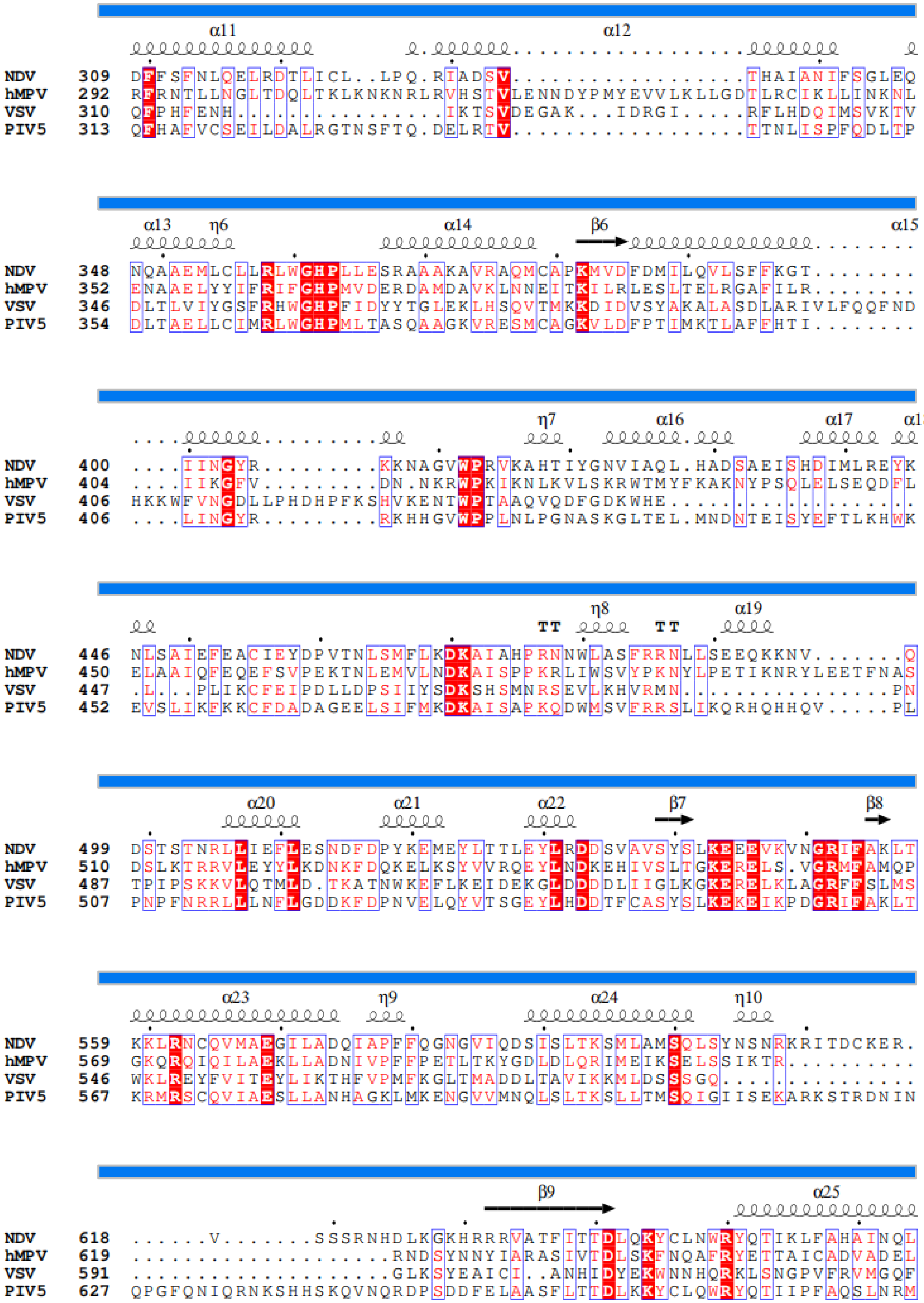

α11 α12
NDV 309 DFFSFNLQELRDTLICL..LPQ.RIADSV..................THAIANIFSGLEQ
hMPV 292 RFRNTLLNGLTDQLTKLKNKNRLRVHSTVLENNDYPMYEVVLKLLGDTLRCIKLLINKNL
VSV 310 QFPHFENH................IKTSVDEGAK...IDRGI.....RFLHDQIMSVKTV
PIV5 313 QFHAFVCSEILDALRGTNSFTQ.DELRTV..................TTNLISPFQDLTP
α13 η6 α14 β6 α15
NDV 348 NQAAEMLCLLRLWGHPLLESRAAAKAVRAQMCAPKMVDFDMILQVLSFFKGT........
hMPV 352 ENAAELYYIFRIFGHPMVDERDAMDAVKLNNEITKILRLESLTELRGAFILR........
VSV 346 DLTLVIYGSFRHWGHPFIDYYTGLEKLHSQVTMKKDIDVSYAKALASDLARIVLFQQFND
PIV5 354 DLTAELLCIMRLWGHPMLTASQAAGKVRESMCAGKVLDFPTIMKTLAFFHTI........
η7 α16 α17 α1
NDV 400 ....IINGYR.........KKNAGVWPRVKAHTIYGNVIAQL.HADSAEISHDIMLREYK
hMPV 404 ....IIKGFV.........DN.NKRWPKIKNLKVLSKRWTMYFKAKNYPSQLELSEQDFL
VSV 406 HKKWFVNGDLLPHDHPFKSHVKENTWPTAAQVQDFGDKWHE...................
PIV5 406 ....LINGYR.........RKHHGVWPPLNLPGNASKGLTEL.MNDNTEISYEFTLKHWK
TT η8 TT α19
NDV 446 NLSAIEFEACIEYDPVTNLSMFLKDKAIAHPRNNWLASFRRNLLSEEQKKNV.......Q
hMPV 450 ELAAIQFEQEFSVPEKTNLEMVLNDKAISPPKRLIWSVYPKNYLPETIKNRYLEETFNAS
VSV 447 .L...PLIKCFEIPDLLDPSIIYSDKSHSMNRSEVLKHVRMN................PN
PIV5 452 EVSLIKFKKCFDADAGEELSIFMKDKAISAPKQDWMSVFRRSLIKQRHQHHQV.....PL
α20 α21 α22 β7 β8
NDV 499 DSTSTNRLLIEFLESNDFDPYKEMEYLTTLEYLRDDSVAVSYSLKEEEVKVNGRIFAKLT
hMPV 510 DSLKTRRVLEYYLKDNKFDQKELKSYVVRQEYLNDKEHIVSLTGKERELS.VGRMFAMQP
VSV 487 TPIPSKKVLQTMLD.TKATNWKEFLKEIDEKGLDDDDLIIGLKGKERELKLAGRFFSLMS
PIV5 507 PNPFNRRLLLNFLGDDKFDPNVELQYVTSGEYLHDDTFCASYSLKEKEIKPDGRIFAKLT
α23 η9 α24 η10
NDV 559 KKLRNCQVMAEGILADQIAPFFQGNGVIQDSISLTKSMLAMSQLSYNSNRKRITDCKER.
hMPV 569 GKQRQIQILAEKLLADNIVPFFPETLTKYGDLDLQRIMEIKSELSSIKTR..........
VSV 546 WKLREYFVITEYLIKTHFVPMFKGLTMADDLTAVIKKMLDSSSGQ...............
PIV5 567 KRMRSCQVIAESLLANHAGKLMKENGVVMNQLSLTKSLLTMSQIGIISEKARKSTRDNIN
β9 α25
NDV 618 ......V.......SSSRNHDLKGKHRRRVATFITTDLQKYCLNWRYQTIKLFAHAINQL
hMPV 619 ....................RNDSYNNYIARASIVTDLSKFNQAFRYETTAICADVADEL
VSV 591 ....GLKSYEAICI..ANHIDYEKWNNHQRKLSNGPVFRVMGQF
PIV5 627 QPGFQNIQRNKSHHSKQVNQRDPSDDFELAASFLTTDLKKYCLQWRYQTIIPFAQSLNRM

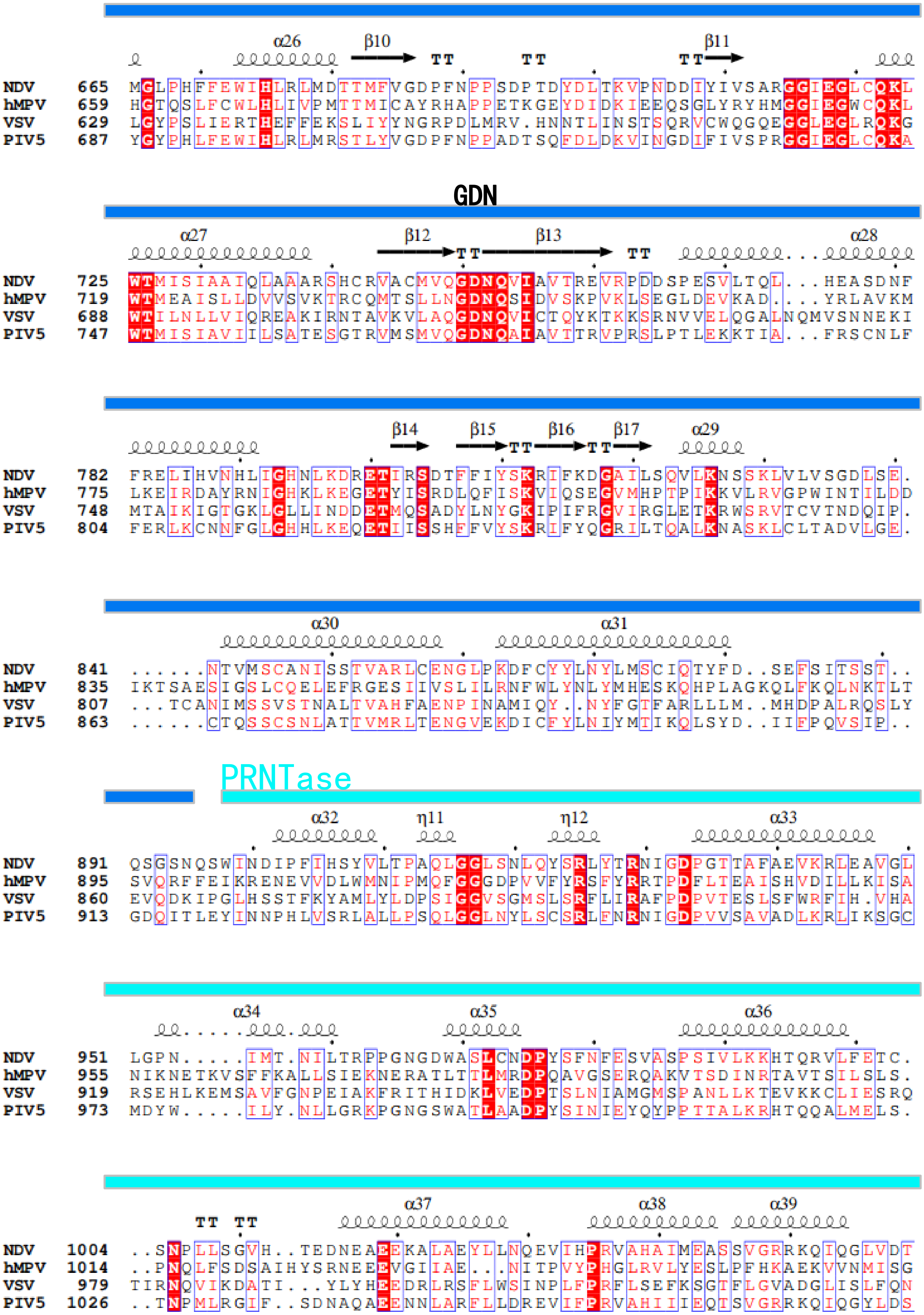

α26 β10 β11
TT TT TT
NDV 665 MGLPHFFEWIHLRLMDTTMFVGDPFNPPSDPTDYDLTKVPNDDIYIVSARGGIEGLCQKL
hMPV 659 HGTQSLFCWLHLIVPMTTMICAYRHAPPETKGEYDIDKIEEQSGLYRYHMGGIEGWCQKL
VSV 629 LGYPSLIERTHEFFEKSLIYYNGRPDLMRV.HNNTLINSTSQRVCWQGQEGGLEGLRQKG
PIV5 687 YGYPHLFEWIHLRLMRSTLYVGDPFNPPADTSQFDLDKVINGDIFIVSPRGGIEGLCQKA
GDN
α27 β12 β13 α28
TT TT
NDV 725 WTMISIAAIQLAAARSHCRVACMVQGDNQVIAVTREVRPDDSPESVLTQL...HEASDNF
hMPV 719 WTMEAISLLDVVSVKTRCQMTSLLNGDNQSIDVSKPVKLSEGLDEVKAD....YRLAVKM
VSV 688 WTILNLLVIQREAKIRNTAVKVLAQGDNQVICTQYKTKKSRNVVELQGALNQMVSNNEKI
PIV5 747 WTMISIAVIILSATESGTRVMSMVQGDNQAIAVTTRVPRSLPTLEKKTIA...FRSCNLF
β14 β15 β16 β17 α29
TT TT
NDV 782 FRELIHVNHLIGHNLKDRETIRSDTFFIYSKRIFKDGAILSQVLKNSSKLVLVSGDLSE.
hMPV 775 LKEIRDAYRNIGHKLKEGETYISRDLQFISKVIQSEGVMHPTPIKKVLRVGPWINTILDD
VSV 748 MTAIKIGTGKLGLLINDDETMQSADYLNYGKIPIFRGVIRGLETKRWSRVTCVTNDQIP.
PIV5 804 FERLKCNNFGLGHHLKEQETIISSHFFVYSKRIFYQGRILTQALKNASKLCLTADVLGE.
α30 α31
NDV 841 ......NTVMSCANISSTVARLCENGLPKDFCYYLNYLMSCIQTYFD..SEFSITSST..
hMPV 835 IKTSAESIGSLCQELEFRGESIIVSLILRNFWLYNLYMHESKQHPLAGKQLFKQLNKTLT
VSV 807 ...TCANIMSSVSTNALTVAHFAENPINAMIQY..NYFGTFARLLLM..MHDPALRQSLY
PIV5 863 ......CTQSSCSNLATTVMRLTENGVEKDICFYLNIYMTIKQLSYD..IIFPQVSIP..
PRNTase
α32 η11 η12 α33
NDV 891 QSGSNQSWINDIPFIHSYVLTPAQLGGLSNLQYSRLYTRNIGDPGTTAFAEVKRLEAVGL
hMPV 895 SVQRFFEIKRENEVVDLWMNIPMQFGGGDPVVFYRSFYRRTPDFLTEAISHVDILLKISA
VSV 860 EVQDKIPGLHSSTFKYAMLYLDPSIGGVSGMSLSRFLIRAFPDPVTESLSFWRFIH.VHA
PIV5 913 GDQITLEYINNPHLVSRLALLPSQLGGLNYLSCSRLFNRNIGDPVVSAVADLKRLIKSGC
α34 α35 α36
NDV 951 LGPN.....IMT.NILTRPPGNGDWASLCNDPYSFNFESVASPSIVLKKHTQRVLFETC.
hMPV 955 NIKNETKVSFFKALLSIEKNERATLTTLMRDPQAVGSERQAKVTSDINRTAVTSILSLS.
VSV 919 RSEHLKEMSAVFGNPEIAKFRITHIDKLVEDPTSLNIAMGMSPANLLKTEVKKCLIESRQ
PIV5 973 MDYW.....ILY.NLLGRKPGNGSWATLAADPYSINIEYQYPPTTALKRHTQQALMELS.
α37 α38 α39
TT TT
NDV 1004 ..SNPLLSGVH..TEDNEAEEKALAEYLLNQEVIHPRVAHAIMEASSVGRRKQIQGLVDT
hMPV 1014 ..PNQLFSDSAIHYSRNEEEVGIIAE...NITPVYPHGLRVLYESLPFHKAEKVVNMISG
VSV 979 TIRNQVIKDATI...YLYHEEDRLRSFLWSINPLFPRFLSEFKSGTFLGVADGLISLFQN
PIV5 1026 ..TNPMLRGIF..SDNAQAEENNLARFLLDREVIFPRVAHIIIEQTSVGRRKQIQGYLDS

```
                α40            α41        α42
NDV   1060  TNTVIKIALSRKPLGIKRLARIINYSSMHAMLFRDDVFLS.......NRAN....HPLVS
hMPV  1069  TKSITNLLQRTSAINGEDIDRAVS......MMLENLGLLSRILSVVVDSIEIPIK...SN
VSV   1036  SRTIRNSFKKK...YHRELDDLIVRSE........VSSLT.......HLGKLHLRRGSCK
PIV5  1082  TRSIMRKSLEIKPLSNRKLNEILDYNINYLA.Y.NLALLK.......NAIEPPTYLKAMT

            TT        α43                         α44  β18        η13
NDV   1109  SDMCSLALADYARNRSWSPLTGGRKILGVSNPDTIELVEGEILSISGGCSKCDSGDEQFT
hMPV  1120  GRLICCQISRTLRETSWNNM....EIVGVTSPSITTCMDV.IYATSS..........HLK
VSV   1078  MWTCSATHADTLRYKSWG.....RTVIGTTVPHPLEMLGP.QHRKETPCAPCNTSGFNYV
PIV5  1133  LETCSIDIARNLRKLSWAPLLGGRNLEGLETPDPIEITAGALIVGSGYCEQCAAGDNRFT

                                         GxxT
            β19   β20                                    TT      α45
NDV   1169  WFHLPSNIELTDDTSKNPPMRVPYLGSKTQERRAASLA.KIAHMSPHVKAALRASSVLIW
hMPV  1165  .GIIIEKFSTDRTTRGQRGPKSPWVGSSTQEKKLVPVY.NRQILSKQQREQLEAIGKMRW
VSV   1132  SVHCPDGIHDV...FSSRGPLPAYLGSKTSESTSILQPWERESKVPLIKRATRLRDAISW
PIV5  1193  WFFLPSGIEIGGDPRDNPPIRVPYIGSRTDERRVASMA.YIRGASSSLKAVLRLAGVYIW

                                                        HR
                     α46              α47
NDV   1228  AYGDNDINWTAALKLARSRCNISSEYLRLLSPLPTAGNLQHRLDDGITQM.TFTPAS.LY
hMPV  1223  VYKGTPGLRRLLNKICLGSLGISYKCVKPLLPRFMSVNFLHRLSVSSRPM.EFPASVPAY
VSV   1189  FVEPDSKLAMTILSNIHSLTGE..EWTKRQHGFKRTGSALHRFSTSRMSHGGFASQSTA.
PIV5  1252  AFGDTLENWIDALDLSHTRVNITLEQLQSLTPLPTSANLTHRLDDGTTTL.KFTPAS.SY

              η14   β21  TT                α48                TT   β22    β23
NDV   1286  RVSPYVH.ISNDSQRLFTEEGVKEGNVVYQQIMLLGLSLIESLFPMTVTKTYDEITLHLH
hMPV  1282  RTTNYHFDTSPINQALSERFGNEDINLVFQNAISCGISIMSVVEQLTGRSPKQ.LVLIPQ
VSV   1246  ALTRLMA.....TTDTMRDLGDQNFDFLFQATLLYAQITTTVA....RDGWITSCTDHYH
PIV5  1310  TFSSFTH.ISNDEQYLTINDKTADSNIIYQQLMITGLGILETWNNPINRTFEESTLHLH

                                                        CD
                TT                                          α49      η15     T
NDV   1345  SKFSCCIREAPVAVPFELTGVAPDLRVVASNKFMYDPNPVAEGDFARLDLAIFKSYELNL
hMPV  1341  .LEEIDIMPPPVFQ..GKF.NYKLVDKITSDQHIFSPDKIDMLTLGKMLMPTIKGQKT..
VSV   1297  IACKSCLRPIEEITLDSSMDYTPP.....DVSHVLKTWRNGEGSWGQEIKQIY.PLEGNW
PIV5  1369  TGASCCVRPVDSCILSEALTVKPHITVPYSNKFVFDEDPLSEYETAKLESLSFQAQLGNI

            T         α50
NDV   1405  ESYSTVELMNI.LSISSGKLIGQSV........VSYDEETSIKNDAIIVYDNTRNWISEA
hMPV  1395  .....DQFLNKRENYFHGNNLIESLSAALACHWCGILTEQCI.ENNIFKKDWGDGFISDH
VSV   1351  KNL.....APAEQSYQVGRCIGFLY.GDLAYRKSTHAEDSSLFPLSIQGRIRGRGFLKGL
PIV5  1429  DAVDMTGKLTL.LSQFTARQIINAI........TGLDESVSLTNDAIVASDYVSNWISEC
```

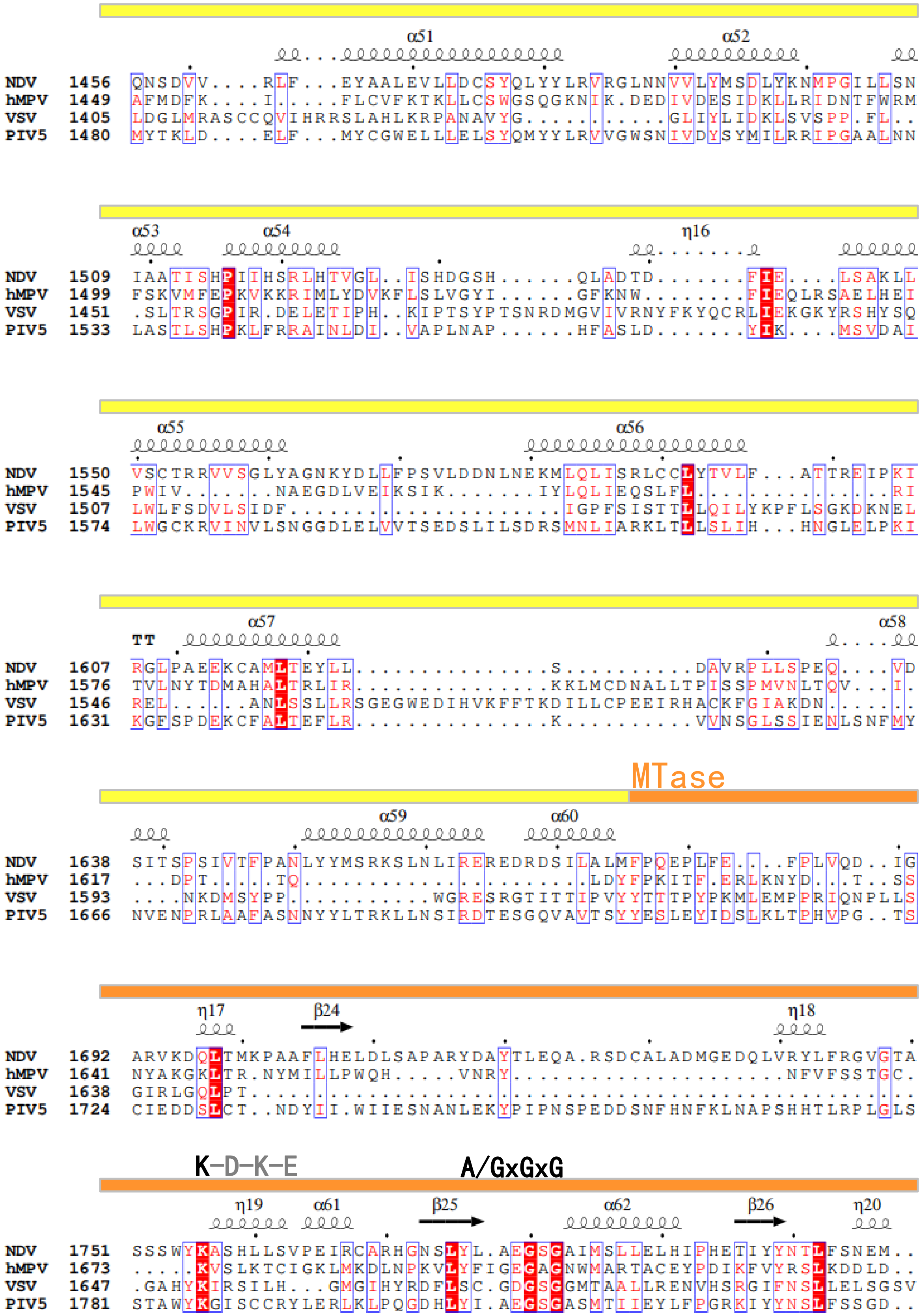

α51 α52
NDV 1456 QNSDVV....RLF...EYAALEVLLDCSYQLYYLRVRGLNNVVLYMSDLYKNMPGILLSN
hMPV 1449 AFMDFK....I.....FLCVFKTKLLCSWGSQGKNIK.DEDIVDESIDKLLRIDNTFWRM
VSV 1405 LDGLMRASCCQVIHRRSLAHLKRPANAVYG.........GLIYLIDKLSVSPP.FL..
PIV5 1480 MYTKLD....ELF...MYCGWELLLELSYQMYYLRVVGWSNIVDYSYMILRRIPGAALNN
α53 α54 η16
NDV 1509 IAATISHPIIHSRLHTVGL..ISHDGSH......QLADTD.......FIE....LSAKLL
hMPV 1499 FSKVMFEPKVKKRIMLYDVKFLSLVGYI......GFKNW........FIEQLRSAELHEI
VSV 1451 .SLTRSGPIR.DELETIPH..KIPTSYPTSNRDMGVIVRNYFKYQCRLIEKGKYRSHYSQ
PIV5 1533 LASTLSHPKLFRRAINLDI..VAPLNAP......HFASLD.......YIK....MSVDAI
α55 α56
NDV 1550 VSCTRRVVSGLYAGNKYDLLFPSVLDDNLNEKMLQLISRLCCLYTVLF...ATTREIPKI
hMPV 1545 PWIV......NAEGDLVEIKSIK.......IYLQLIEQSLFL..............RI
VSV 1507 LWLFSDVLSIDF.......................IGPFSISTTLLQILYKPFLSGKDKNEL
PIV5 1574 LWGCKRVINVLSNGGDLELVVTSEDSLILSDRSMNLIARKLTLLSLIH...HNGLELPKI
TT α57 α58
NDV 1607 RGLPAEEKCAMLTEYLL...............S..........DAVRPLLSPEQ....VD
hMPV 1576 TVLNYTDMAHALTRLIR...............KKLMCDNALLTPISSPMVNLTQV...I.
VSV 1546 REL......ANLSSLLRSGEGWEDIHVKFFTKDILLCPEEIRHACKFGIAKDN.......
PIV5 1631 KGFSPDEKCFALTEFLR...............K..........VVNSGLSSIENLSNFMY
MTase
α59 α60
NDV 1638 SITSPSIVTFPANLYYMSRKSLNLIREREDRDSILALMFPQEPLFE....FPLVQD..IG
hMPV 1617 ...DPT....TQ.............LDYFPKITF.ERLKNYD...T..SS
VSV 1593 ....NKDMSYPP..........WGRESRGTITTIPVYYTTTPYPKMLEMPPRIQNPLLS
PIV5 1666 NVENPRLAAFASNNYYLTRKLLNSIRDTESGQVAVTSYYESLEYIDSLKLTPHVPG..TS
η17 β24 η18
NDV 1692 ARVKDQLTMKPAAFLHELDLSAPARYDAYTLEQA.RSDCALADMGEDQLVRYLFRGVGTA
hMPV 1641 NYAKGKLTR.NYMILLPWQH.....VNRY..................NFVFSSTGC.
VSV 1638 GIRLGQLPT......................................
PIV5 1724 CIEDDSLCT..NDYII.WIIESNANLEKYPIPNSPEDDSNFHNFKLNAPSHHTLRPLGLS
K-D-K-E
A/GxGxG
η19 α61 β25 α62 β26 η20
NDV 1751 SSSWYKASHLLSVPEIRCARHGNSLYL.AEGSGAIMSLLELHIPHETIYYNTLFSNEM..
hMPV 1673 .....KVSLKTCIGKLMKDLNPKVLYFIGEGAGNWMARTACEYPDIKFVYRSLKDDLD..
VSV 1647 .GAHYKIRSILH...GMGIHYRDFLSC.GDGSGGMTAALLRENVHSRGIFNSLLELSGSV
PIV5 1781 STAWYKGISCCRYLERLKLPQGDHLYI.AEGSGASMTIIEYLFPGRKIYYNSLFSSGD..

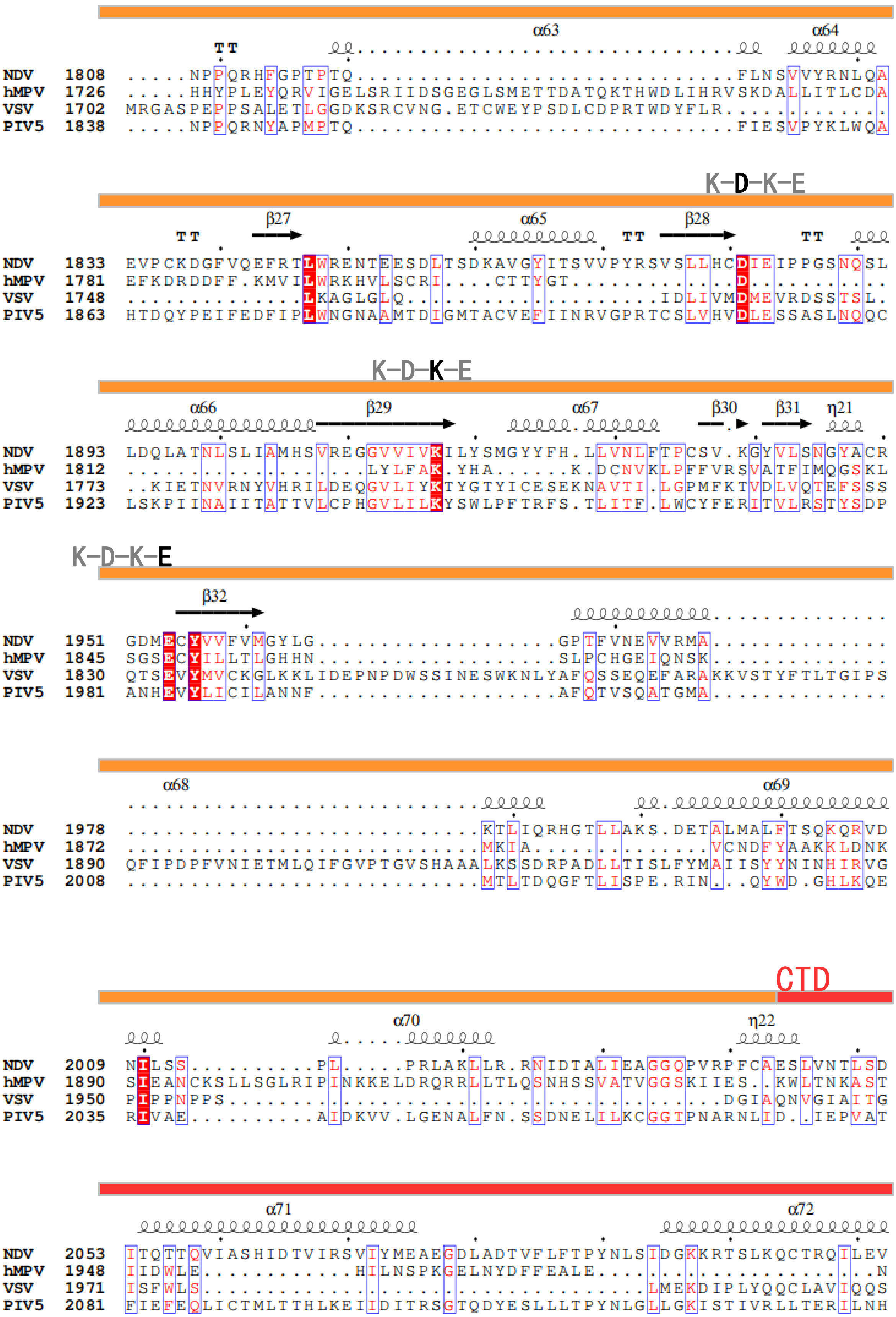

α63
α64
TT
NDV 1808 .....NPPQRHFGPTPTQ.............................FLNSVVYRNLQA
hMPV 1726 .....HHYPLEYQRVIGELSRIIDSGEGLSMETTDATQKTHWDLIHRVSKDALLITLCDA
VSV 1702 MRGASPEPPSALETLGGDKSRCVNG.ETCWEYPSDLCDPRTWDYFLR.............
PIV5 1838 .....NPPQRNYAPMPTQ.............................FIESVPYKLWQA
K-D-K-E
TT
β27
α65
TT
β28
TT
NDV 1833 EVPCKDGFVQEFRTLWRENTEESDLTSDKAVGYITSVVPYRSVSLLHCDIEIPPGSNQSL
hMPV 1781 EFKDRDDFF.KMVILWRKHVLSCRI....CTTYGT...........D.............
VSV 1748 ..............LKAGLGLQ.................IDLIVMDMEVRDSSTSL.
PIV5 1863 HTDQYPEIFEDFIPLWNGNAAMTDIGMTACVEFIINRVGPRTCSLVHVDLESSASLNQQC
K-D-K-E
α66
β29
α67
β30
β31
η21
NDV 1893 LDQLATNLSLIAMHSVREGGVVIVKILYSMGYYFH.LLVNLFTPCSV.KGYVLSNGYACR
hMPV 1812 ..................LYLFAK.YHA......K.DCNVKLPFFVRSVATFIMQGSKL
VSV 1773 ..KIETNVRNYVHRILDEQGVLIYKTYGTYICESEKNAVTI.LGPMFKTVDLVQTEFSSS
PIV5 1923 LSKPIINAIITATTVLCPHGVLILKYSWLPFTRFS.TLITF.LWCYFERITVLRSTYSDP
K-D-K-E
β32
NDV 1951 GDMECYVVFVMGYLG...................GPTFVNEVVRMA..............
hMPV 1845 SGSECYILLTLGHHN...................SLPCHGEIQNSK..............
VSV 1830 QTSEVYMVCKGLKKLIDEPNPDWSSINESWKNLYAFQSSEQEFARAKKVSTYFTLTGIPS
PIV5 1981 ANHEVYLICILANNF...................AFQTVSQATGMA..............
α68
α69
NDV 1978 ...........................KTLIQRHGTLLAKS.DETALMALFTSQKQRVD
hMPV 1872 ....MKIA..............VCNDFYAAKKLDNK
VSV 1890 QFIPDPFVNIETMLQIFGVPTGVSHAAALKSSDRPADLLTISLFYMAIISYYNINHIRVG
PIV5 2008 ...........................MTLTDQGFTLISPE.RIN...QYWD.GHLKQE
CTD
α70
η22
NDV 2009 NILSS..........PL.....PRLAKLLR.RNIDTALIEAGGQPVRPFCAESLVNTLSD
hMPV 1890 SIEANCKSLLSGLRIPINKKELDRQRRLLTLQSNHSSVATVGGSKIIES..KWLTNKAST
VSV 1950 PIPPNPPS.........................DGIAQNVGIAITG
PIV5 2035 RIVAE..........AIDKVV.LGENALFN.SSDNELILKCGGTPNARNLID..IEPVAT
α71
α72
NDV 2053 ITQTTQVIASHIDTVIRSVIYMEAEGDLADTVFLFTPYNLSIDGKKRTSLKQCTRQILEV
hMPV 1948 IIDWLE...........HILNSPKGELNYDFFEALE......................N
VSV 1971 ISFWLS.......................LMEKDIPLYQQCLAVIQQS
PIV5 2081 FIEFEQLICTMLTTHLKEIIDITRSGTQDYESLLLTPYNLGLLGKISTISVRLLTERILNH

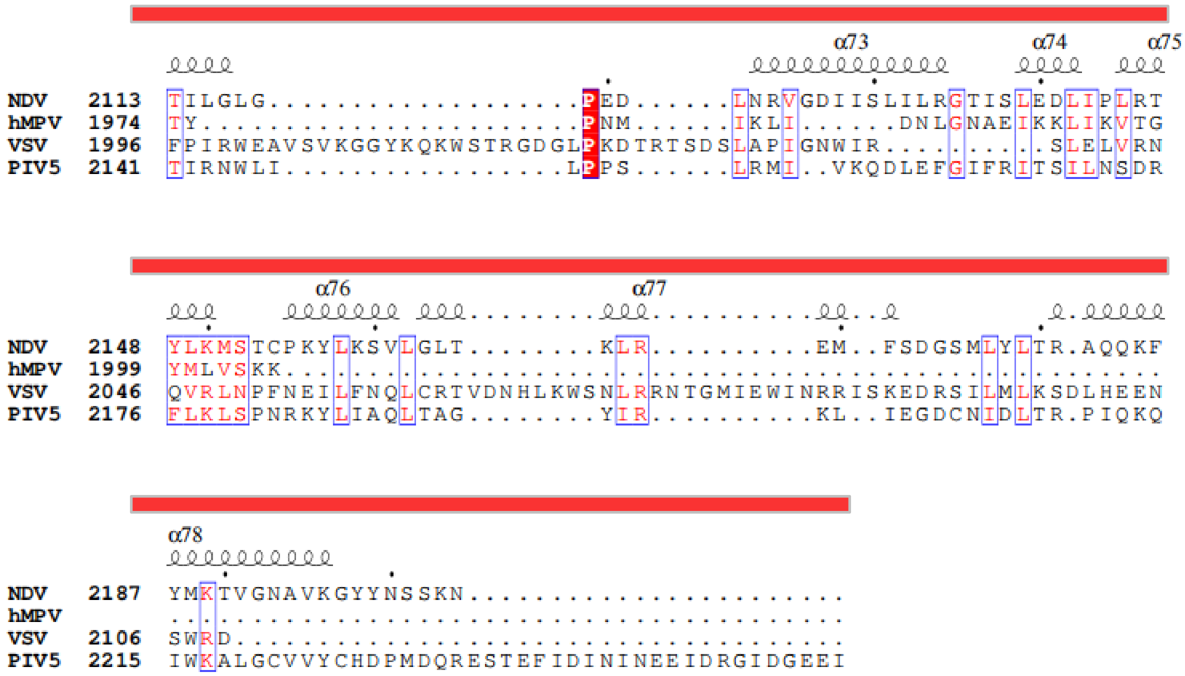

Supplementary Figure 2: Sequence alignment of NDV L proteins with PIV5, hMPV and VSV. Sequence alignment for NDV, PIV5, hMPV and VSV L protein. Colored bars above sequence alignment shows domain boundaries. Functional motifs are marked above the sequence.

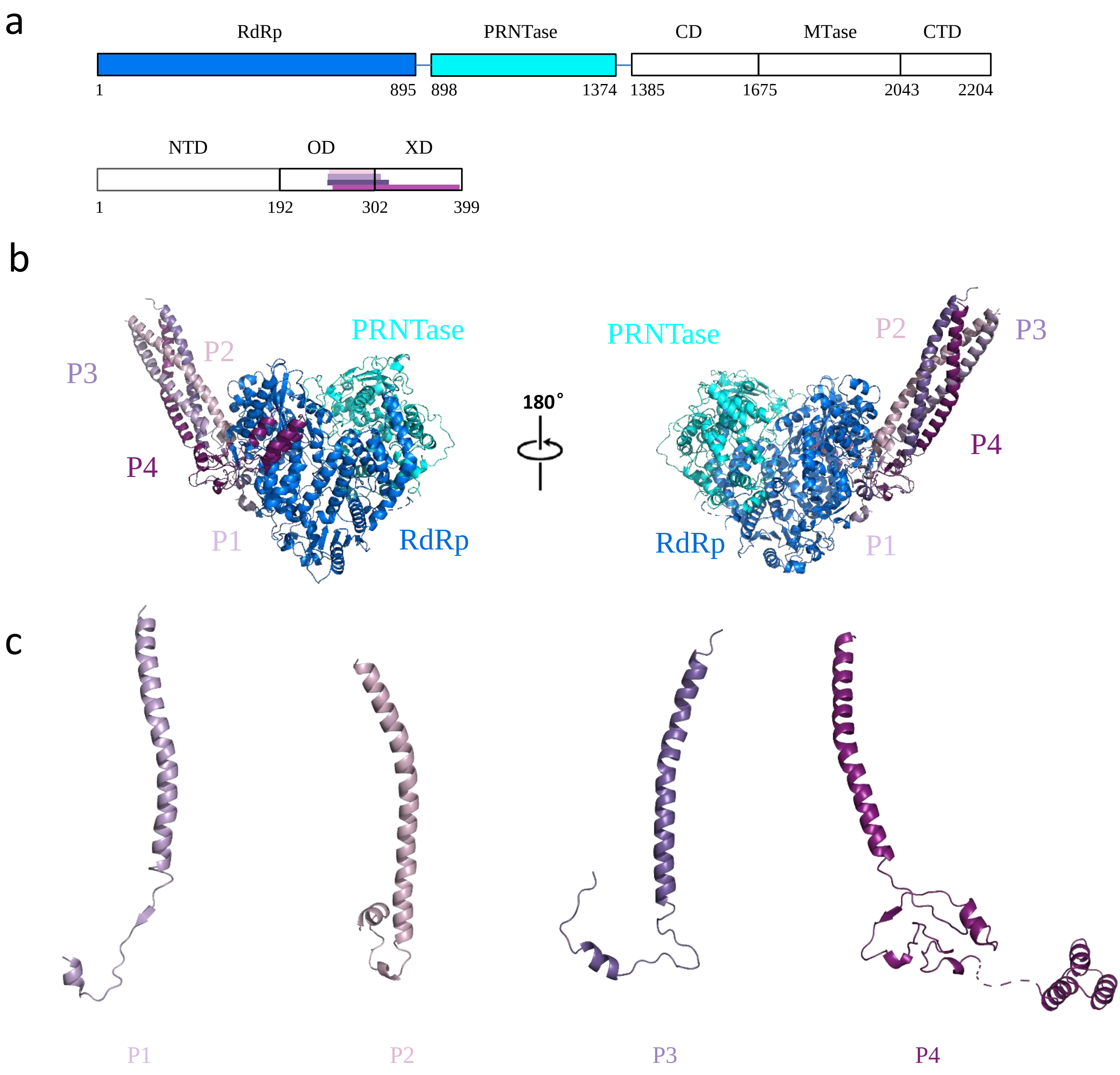

Supplementary Figure 3: Overall structure of NDV L_c-P_e complex. a, Schematic diagram of the domain architecture of NDV L protein divided into five parts: RdRp, PRNTase, CD, MTase and CTD. NDV P protein is divided into three parts: NTD, OD and XD. RdRp and PRNTase domains of NDV L is represented by blue and cyan; P protein monomers is represented using unique colors. **b,** Cartoon representation of NDV L_c-P_e complex. The structures are colored by domains, the coloring scheme is identical to that in (a). The unresolved linker regions are connected by dashed lines. The right region is rotated 180° along the vertical axis. **c,** Structures adopted by the four individual P subunits bound to L.

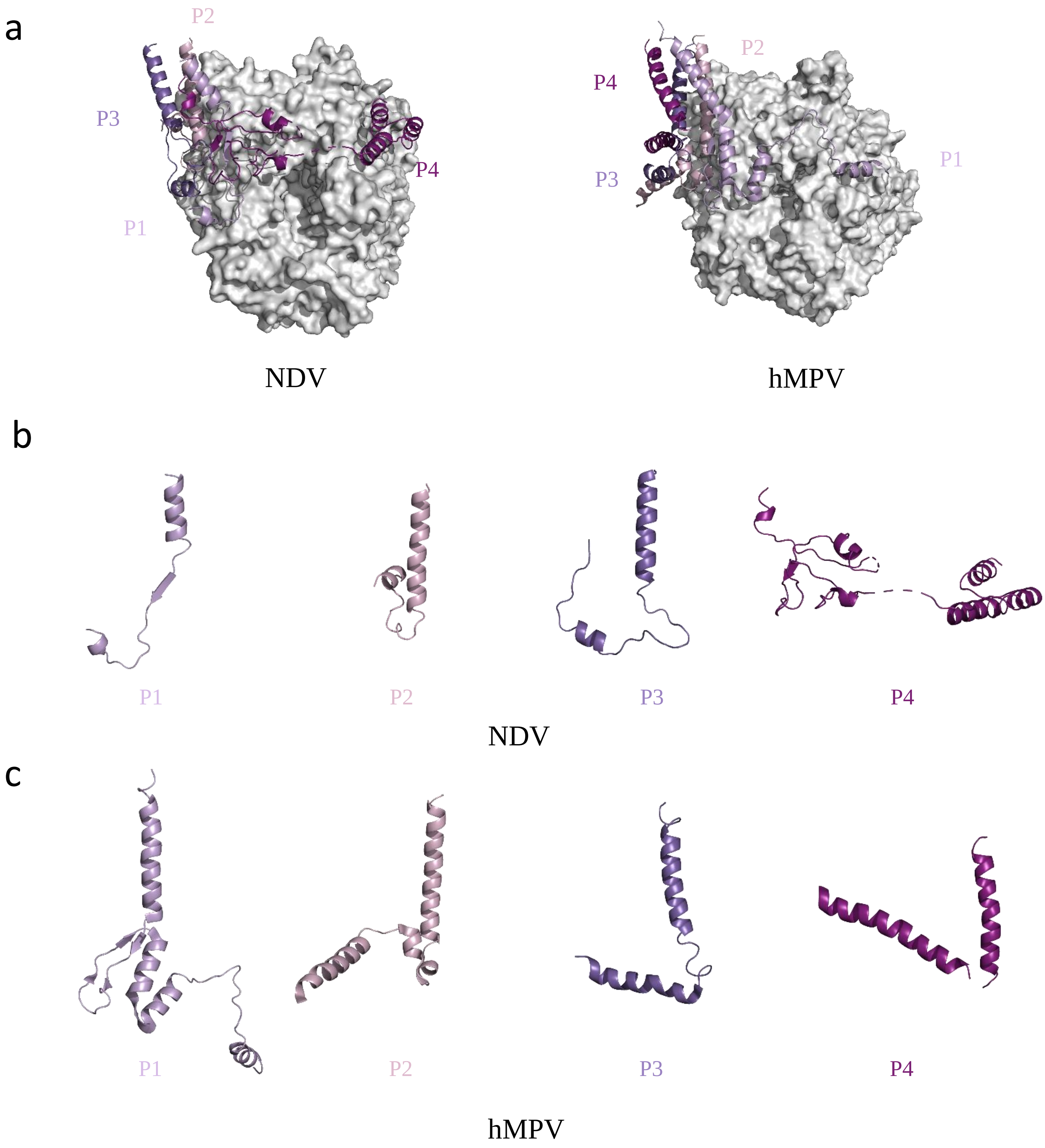

Supplementary Figure 4: P protein tetramer in complex with L. a, NDV (left) and hMPV (right) L proteins are shown as a gray molecular surface, and P protein monomers are shown in cartoon. Each P protein is represented using a unique color. **b**,**c,** Structures adopted by the four individual P protein subunits bound to L, in complex structures of NDV (b) and hMPV (c).

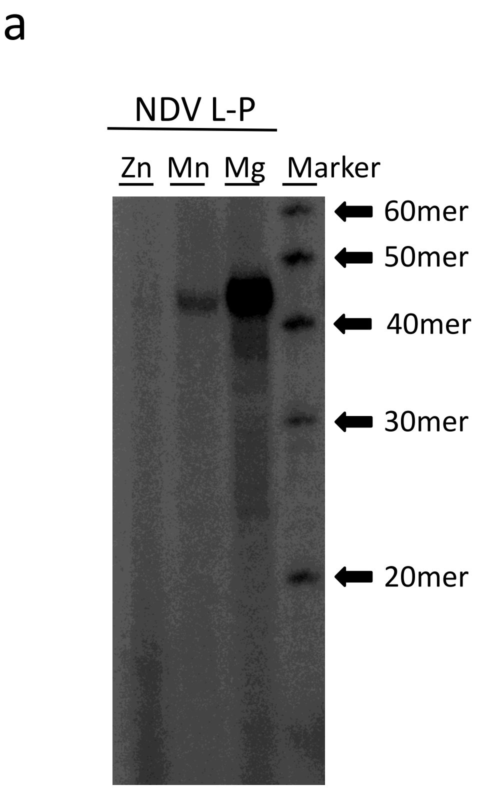

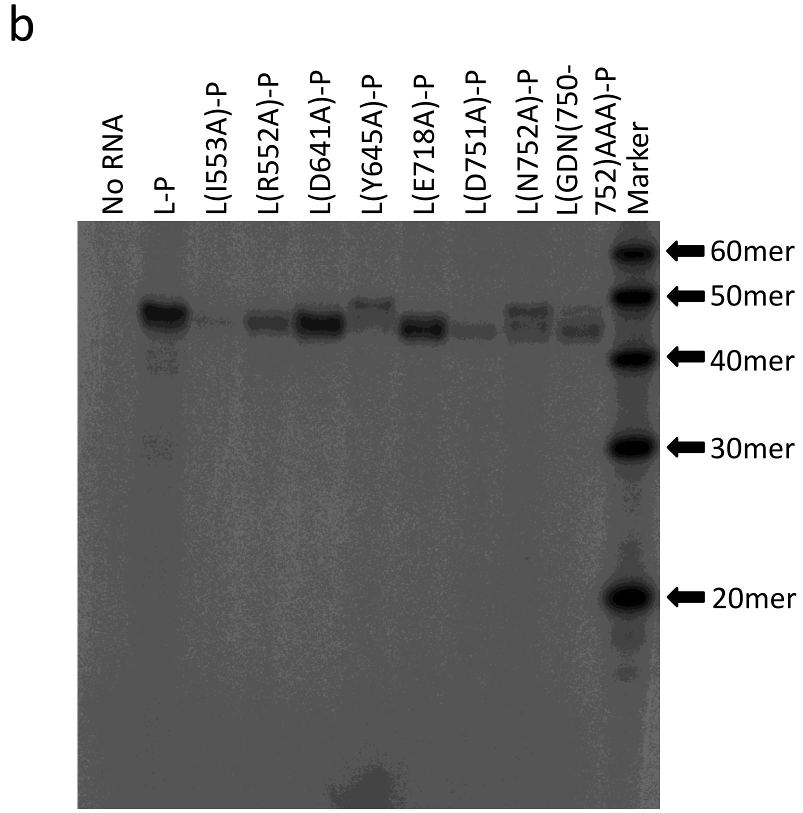

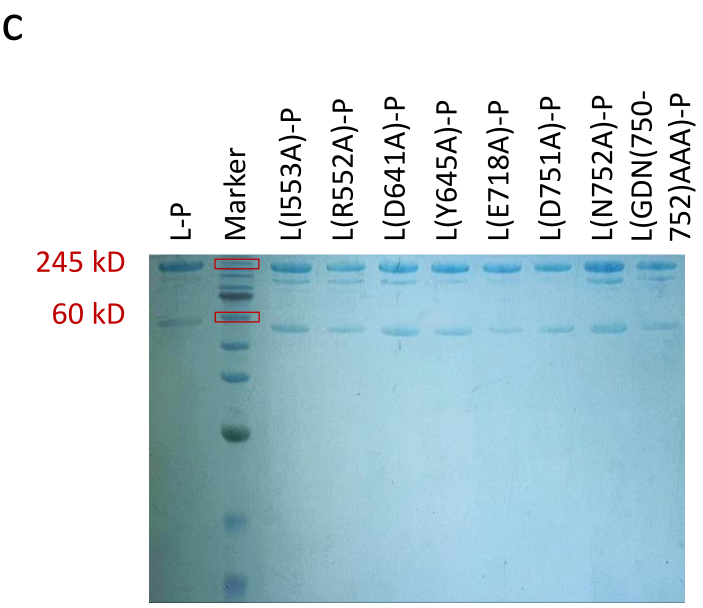

Supplementary Figure 5: *In vitro* functional assays for NDV L-P complex. a, Show the effect of metal ions on enzymatic activity, where Mg^{2+} and Mn^{2+} promote RNA replication activity, while Zn^{2+} suppresses the activity of L protein. Final concentration for Mg^{2+}, Mn^{2+} and Zn^{2+} were 5 mM in the assay. **b,** The RNA synthesis reactions show the purified native L-P complex and its mutants' activity. **c**, SDS-PAGE was performed to assess the quality of L-P complex mutants. Source data are provided as a Source Data file. Sample preparation-related experiments including protein purification and enzymatic assays were reproduced at least twice independently.

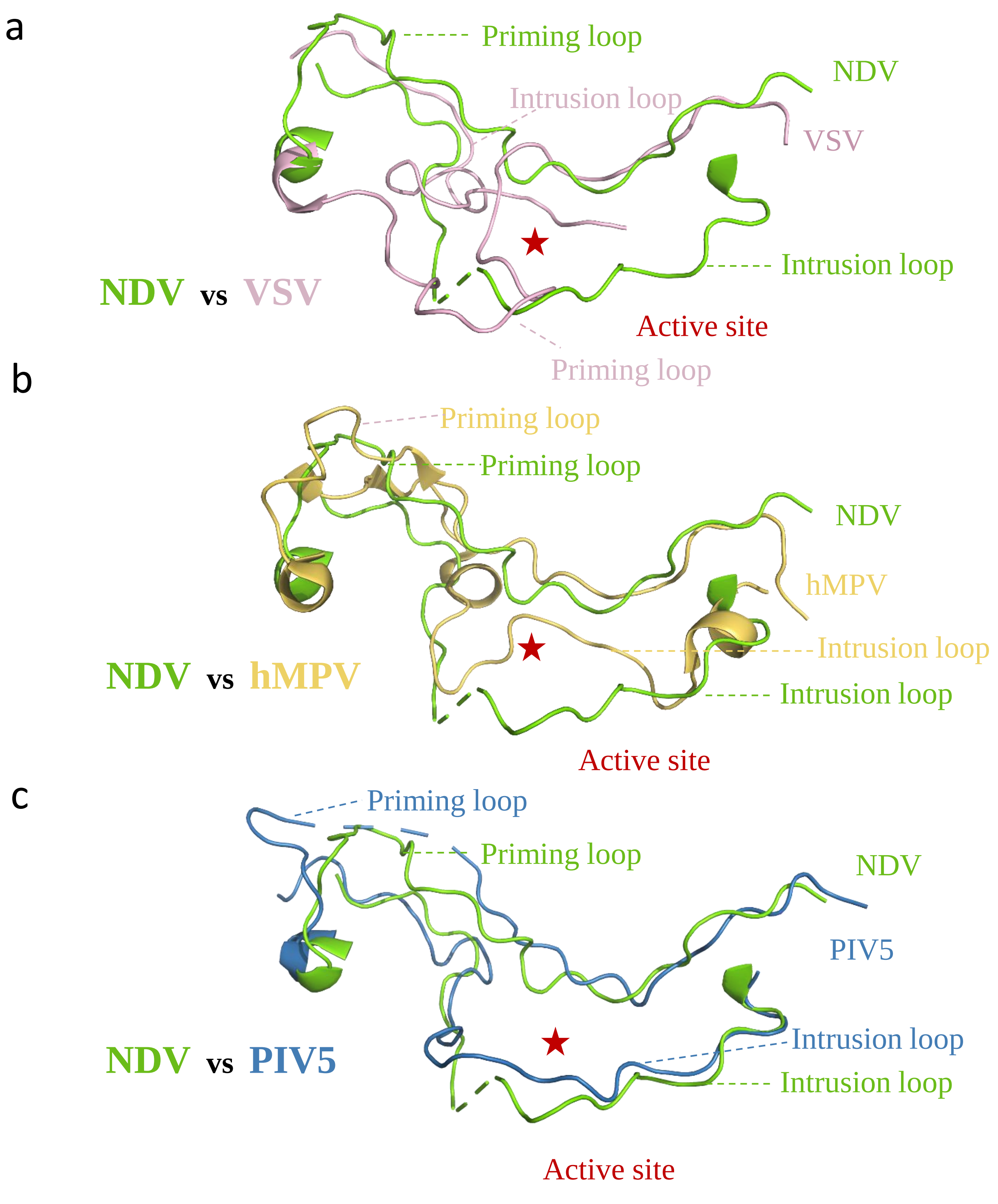

Supplementary Fig 6: Comparison of NDV priming loop and intrusion loop with related viruses. a,b,c, The comparison of NDV priming loop and intrusion loop with VSV (a), hMPV (b) and PIV5 (c) respectively. The catalytic site is indicated by a red star.

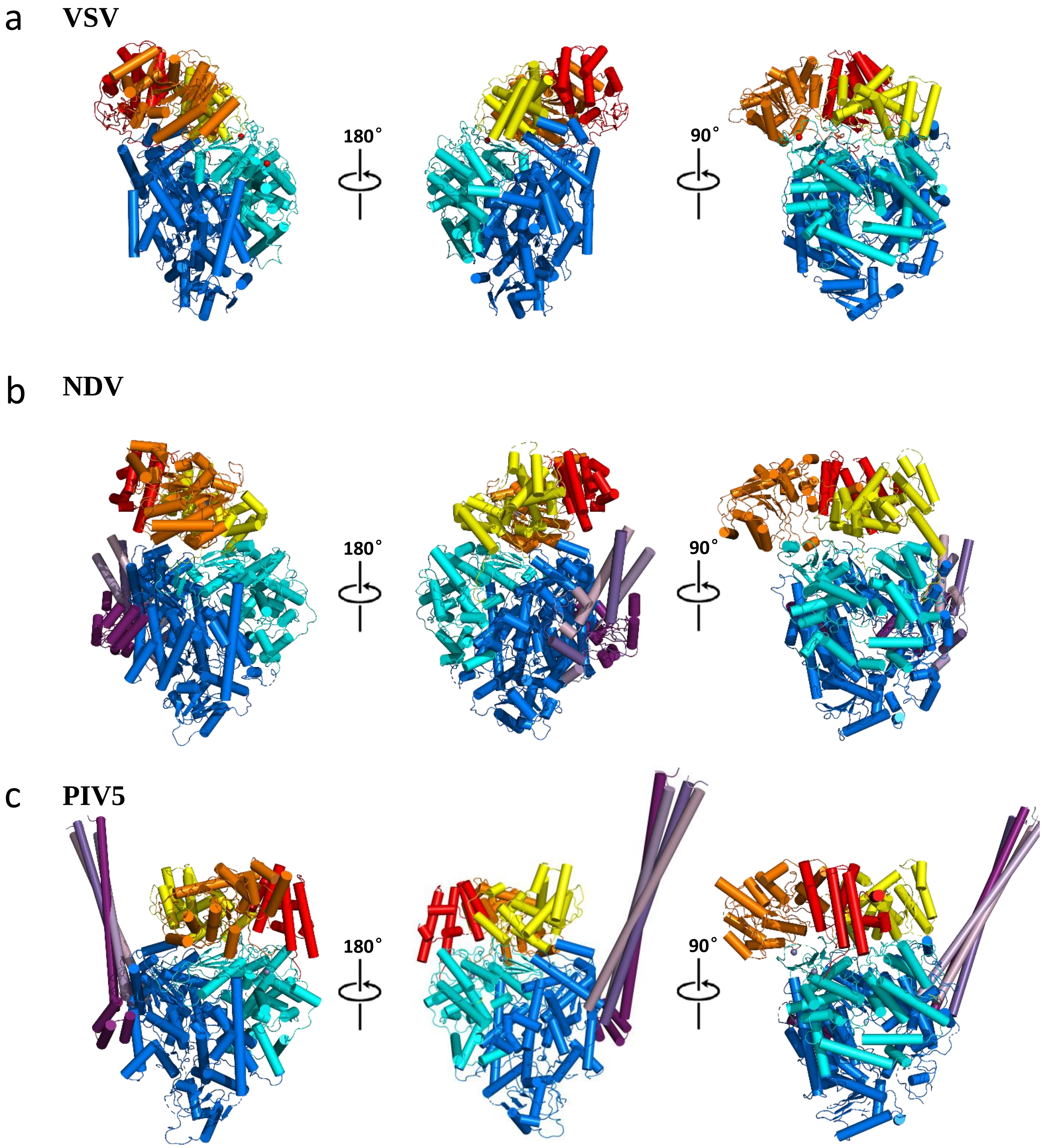

Supplementary Figure 7: C-terminal conformations in the structures of nsNSV L-P complex. a,b,c, L-P complex structures of VSV (a), NDV (b) and PIV5 (c) are shown in cartoon and colored by domains, with α-helix shown as cylinders. Structures representation in each panel are shown in the same orientation after superimposing RdRp domain and PRNTase domain.

Supplementary Table 1: Statistics of Data Collection, Image Processing and Model Building.

**Data collection**			
	**L_c-P**	**L_f-P**	**L_c-P_e**
**EM equipment**	FEI Titan Krios	FEI Titan Krios	FEI Titan Krios
**Voltage (kV)**	300	300	300
**Detector**	Gatan K2	Gatan K2	Gatan K2
**Pixel size (Å/pixel)**	1.04	1.04	1.04
**Electron dose (e^-/Å2)**	60	60	60
**Defocus range (μm)**	-1.2-2.5	-1.2-2.5	-1.2-2.5
**Reconstruction**			
**Software**	Relion3.0	Relion3.0	Relion3.0
**Number of used particles**	455k	42.5k	153k
**Symmetry**	C1	C1	C1
**Map sharpening B-factor ($Å^2$)**	-134.7	-70	-120.6
**Final resolution (Å)**	3.0	3.41	3.25
**Model building**			
**Software**	Coot	Coot	Coot
**Model Refinement**			
**Software**	PHENIX	PHENIX	PHENIX
**Map CC (mask)**	0.815	0.816	0.745
**Map CC (peaks)**	0.615	0.697	0.640
**Map CC (volume)**	0.777	0.809	0.731
**Rmsd (bonds) (Å)**	0.0092	0.0087	0.0114
**Rmsd (angles) (º)**	1.33	1.27	1.41
**Model composition**			
**L Protein residues**	1309	2059	1310
**P Protein residues**	242	262	373
**Validation**			
**MolProbity score**	1.52	1.72	1.87
**Clash score**	4.58	5.17	6.46
**Ramachandran plot**			
**Outliers (%)**	0	0	0
**Allowed (%)**	4.2	6.94	7.67
**Favored (%)**	95.8	93.06	92.33
**Rotamer outliers (%)**	0.66	0.34	1.14
**Cβ outliers (%)**	0	0	0

Supplementary Table 2: Summary of the model.

Subunit Name	Chain	Total residues/ range built	Unmodelled residues	% atomic model
		L_c-P complex		
L	A	2204/8-544, 553-583, 588-611, 629-887, 894-1194， 1209-1265, 1278-1302, 1310-1384	1-7, 545-552, 584-587, 612-628, 888-893, 1195-1208, 1266-1277, 1303-1309, 1385-2204	1309/2204
P1	B	399/268-301	1-267, 302-399	34/399
P2	C	399/264-305	1-263, 306-399	42/399
P3	D	399/262-312,	1-261, 313-399	51/399
P4	E	399/274-289, 294-342, 350-399	1-273, 290-293, 343-349	115/399
		L_f-P complex		
L	A	2204/8-544, 553-583, 587-610, 629-889, 894-1268, 1277-1304, 1310-1431, 1442-1458, 1461-1573, 1579-1597, 1601-1625, 1634-1641, 1645-1676, 1696-1718, 1737-2048, 2053-2076, 2094-2201	1-7, 545-552, 584-586, 611-628, 890-893, 1269-1276, 1305-1309, 1432-1441, 1459-1460, 1574-1578, 1598-1600, 1626-1633, 1642-1644, 1677-1685, 1719-1736, 2049-2052, 2077-2093, 2202-2204	2059/2204
P1	B	399/261-301	1-260, 302-399	41/399
P2	C	399/259-305	1-258, 306-399	47/399
P3	D	399/259-312	1-258, 313-399	54/399
P4	E	399/274-343, 350-399	1-273, 344-349	120/399
		L_c-P_e complex		
L	A	2204/8-544, 553-583, 588-611, 629-888, 894-1194, 1209-1265, 1278-1302, 1310-1384	1-7, 545-552, 584-587, 612-628, 889-893, 1195-1208, 1266-1277, 1303-1309, 1385-2204	1310/2204
P1	B	399/234-301	1-233, 302-399	68/399
P2	C	399/236-306	1-235, 307-399	71/399
P3	D	399/236-311	1-235, 312-399	76/399
P4	E	399/236-343, 350-399	1-235, 344-349	158/399

Supplementary Table 3: RNA oligonucleotides used for *in vitro* functional assays

vRNA	
3'-10nt	5’-UCUGUUUGGU-3’
3'-20nt	5’-UCACAGAAUCUCUGUUUGGU-3’
3'-30nt	5’-GUAUCGUACCUCACAGAAUCUCUGUUUGGU-3’
3'-40nt	5’-CCAUCGCCUUGUAUCGUACCUCACAGAAUCUCUGUUUGGU-3’
3'-50nt	5’-UUCGAUUGCUCCAUCGCCUUGUAUCGUACCUCACAGAAUCUC UGUUUGGU-3’
3'-60nt	5’-CCCGUGCGAUUUCGAUUGCUCCAUCGCCUUGUAUCGUACCUC ACAGAAUCUCUGUUUGGU-3’
**cRNA**	
3'-30nt	5’-AUUCUGAUAUUCACCAAAUCUUUGUUUGGU-3’
3'-40nt	5’-UUGUCACAUUAUUCUGAUAUUCACCAAAUCUUUGUUUGGU-3’
3'-50nt	5’-ACAGAAUAGCUUGUCACAUUAUUCUGAUAUUCACCAAAUCUU UGUUUGGU-3’
