# Supplementary figures and images for: Structure of the Newcastle Disease Virus L protein in complex with tetrameric phosphoprotein
Source: Nat Commun. 2023 Mar 10;14:1324. doi: 10.1038/s41467-023-37012-y (PMC10006412; doi:10.1038/s41467-023-37012-y)

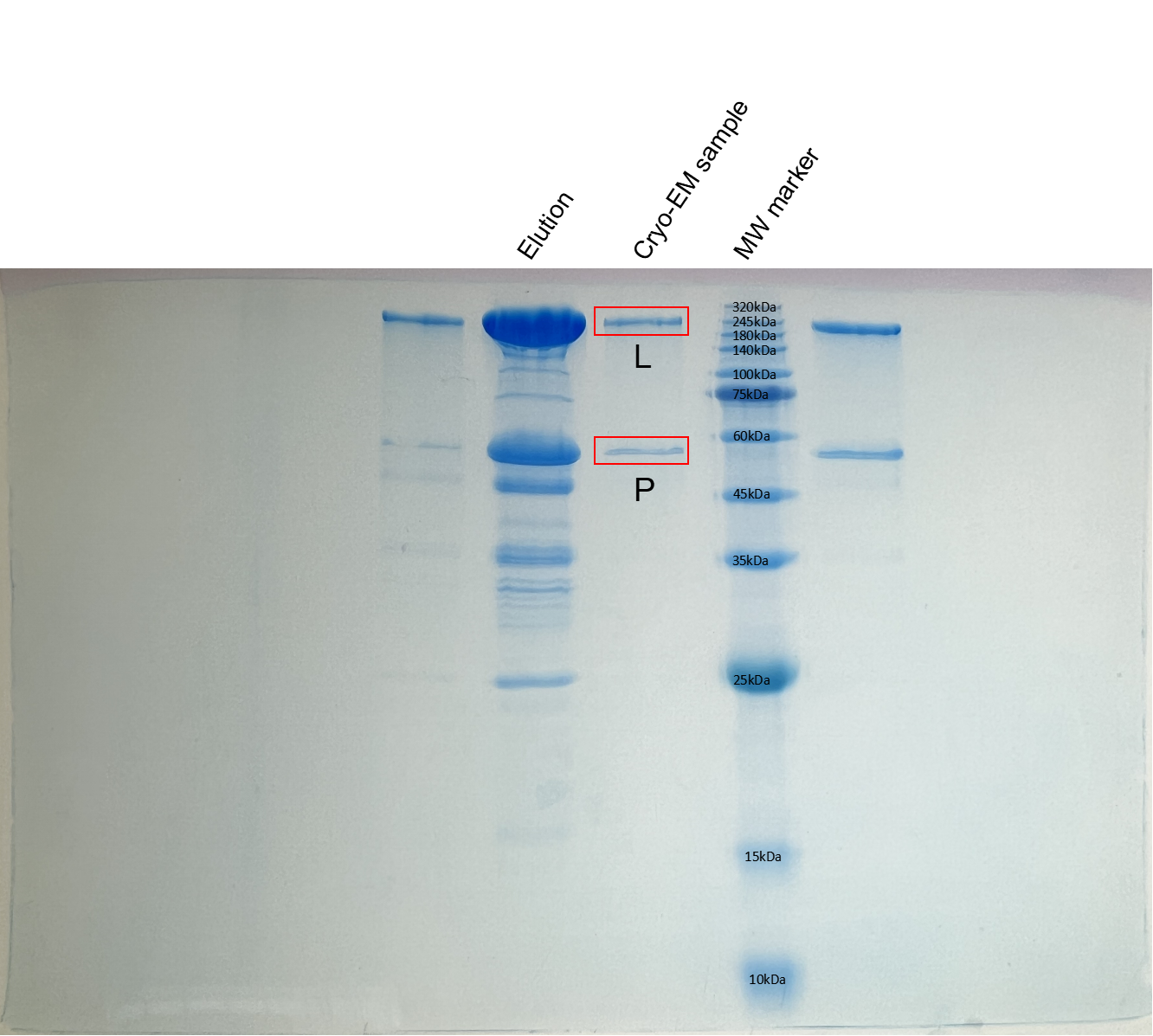

Supplement: Supplementary file 4 — Source Data [file 41467_2023_37012_MOESM4_ESM.zip › Figure 1a-source data.png]

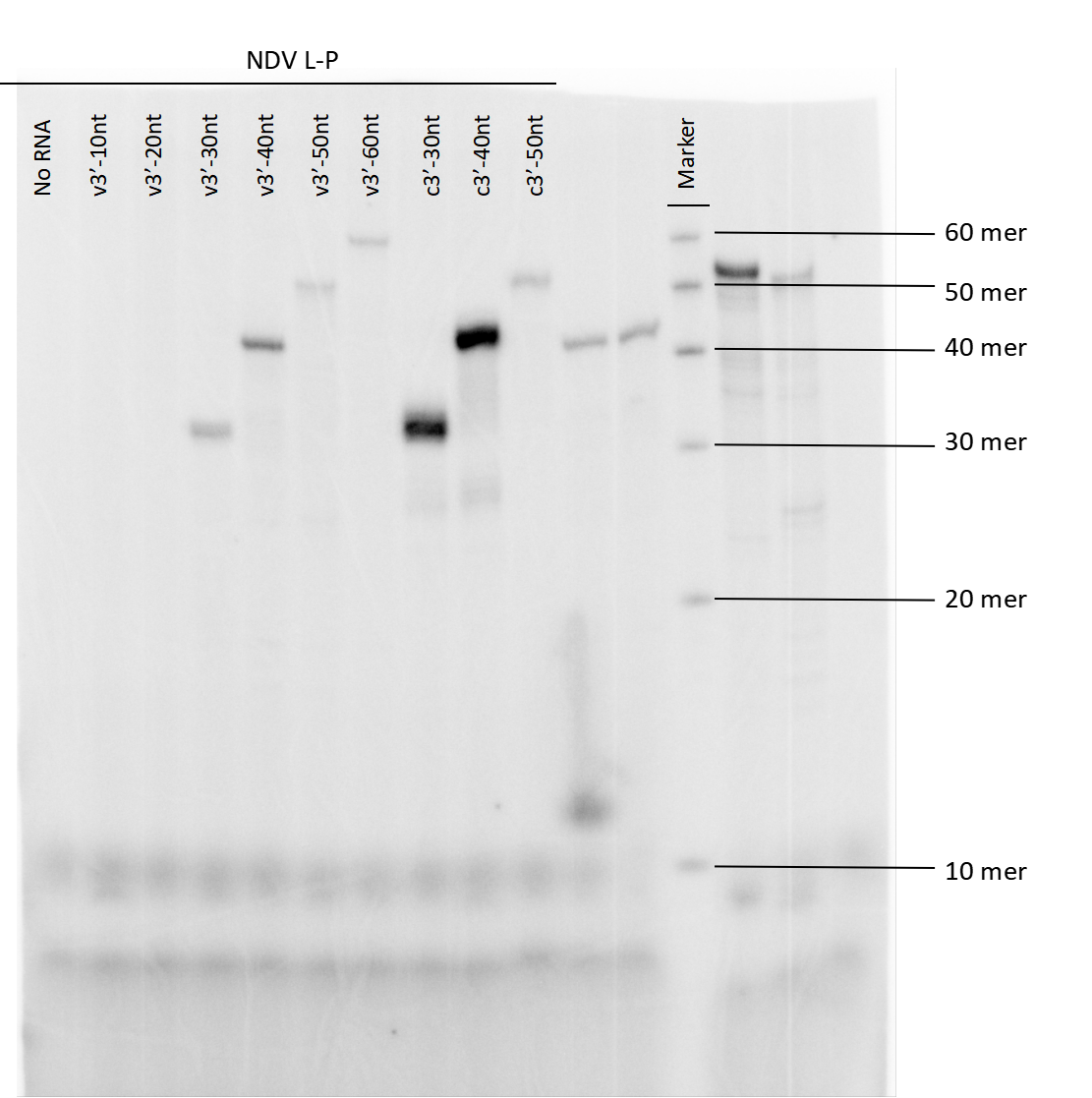

Supplement: Supplementary file 4 — Source Data [file 41467_2023_37012_MOESM4_ESM.zip › Figure 1b-source data.png]

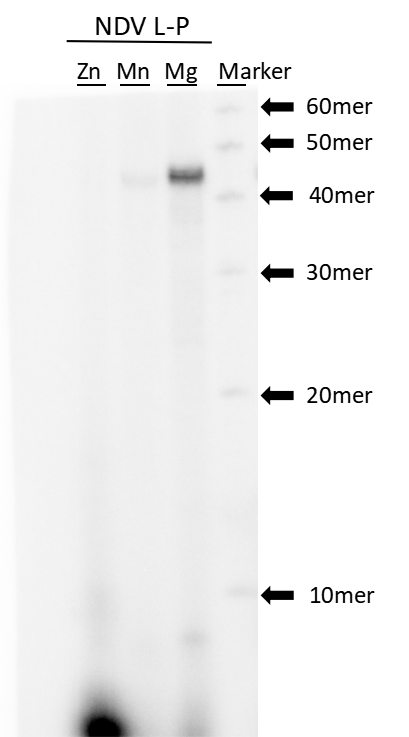

Supplement: Supplementary file 4 — Source Data [file 41467_2023_37012_MOESM4_ESM.zip › Supplementary Figure 5a-source data.png]

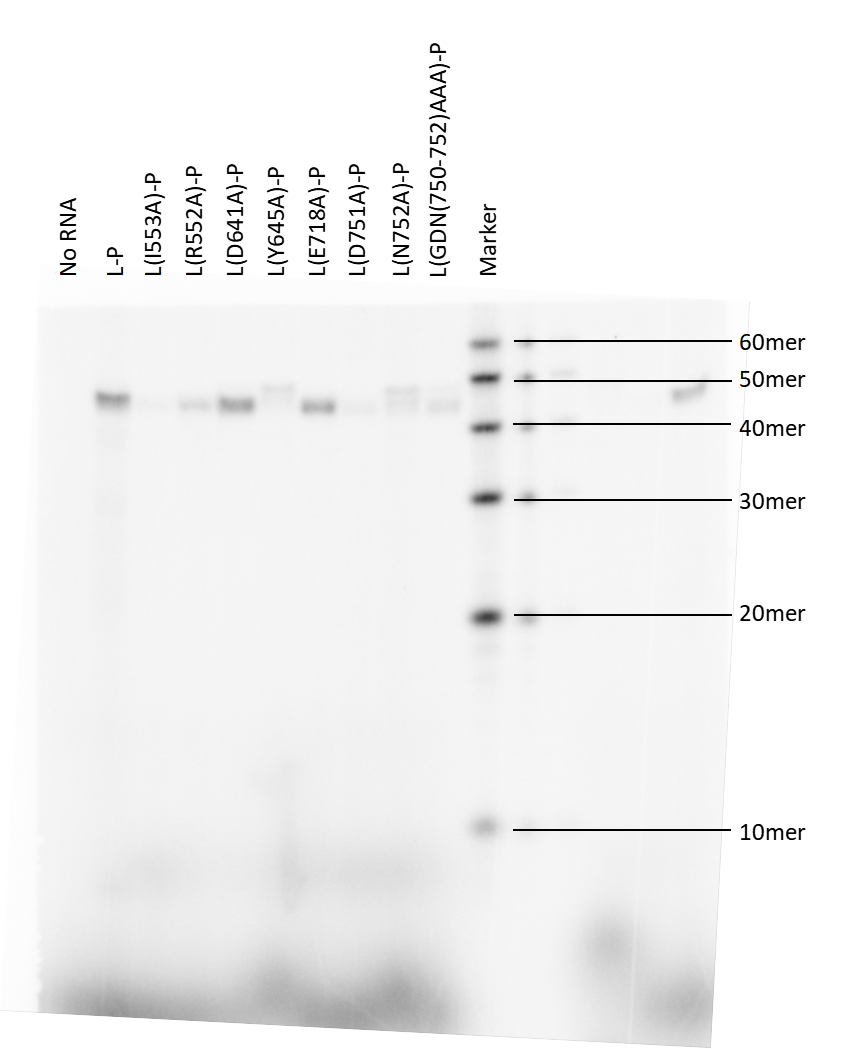

Supplement: Supplementary file 4 — Source Data [file 41467_2023_37012_MOESM4_ESM.zip › Supplementary Figure 5b-source data.png]

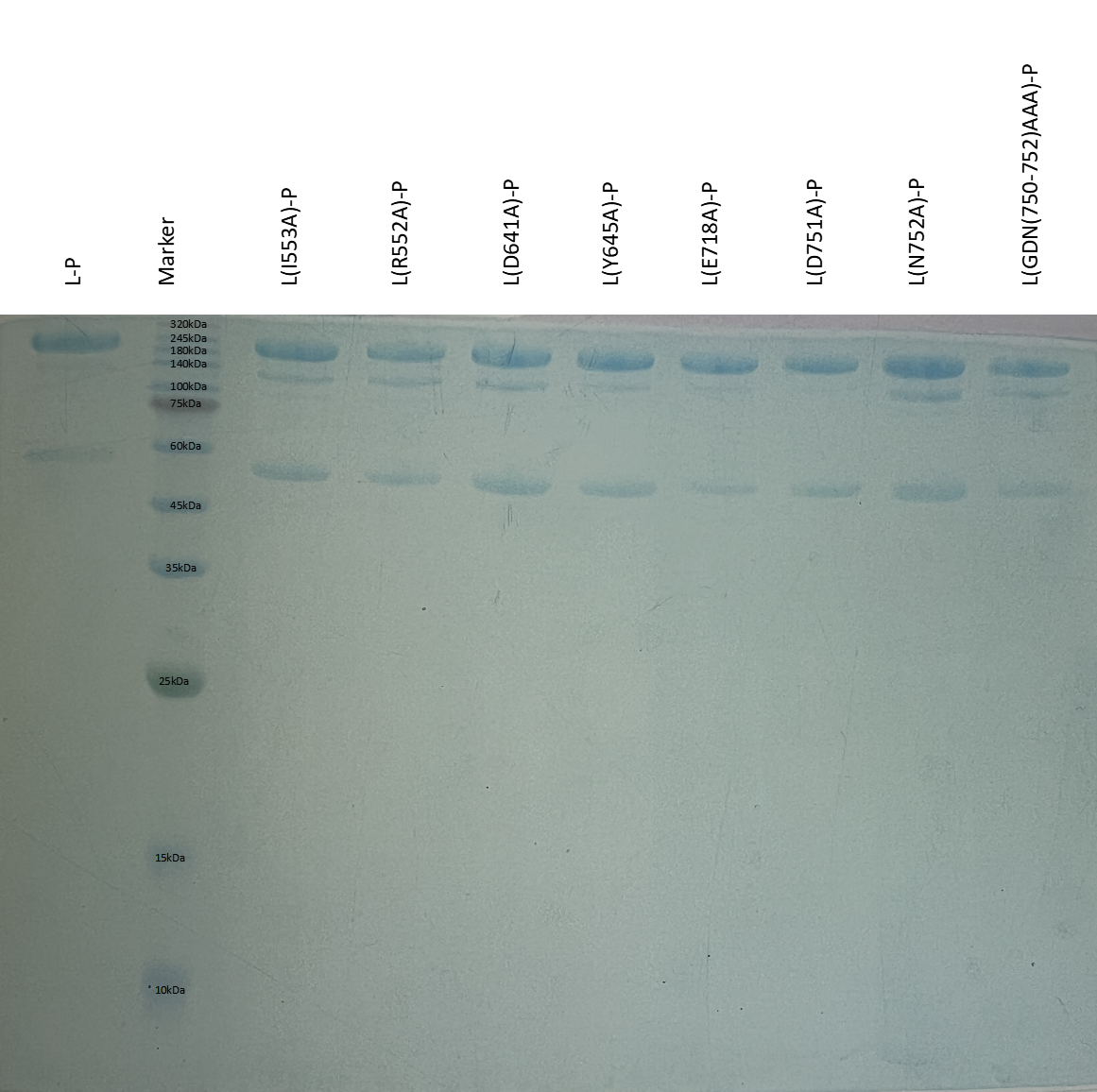

Supplement: Supplementary file 4 — Source Data [file 41467_2023_37012_MOESM4_ESM.zip › Supplementary Figure 5c-source data.tif]
